# Three-Dimensional Textile Platform for Electrochemical Devices and its Application to Dye-Sensitized Solar Cells

**DOI:** 10.1038/s41598-018-38426-1

**Published:** 2019-02-20

**Authors:** Min Ju Yun, Yeon Hyang Sim, Seung I. Cha, Seon Hee Seo, Dong Yoon Lee

**Affiliations:** 1 Energy Conversion Research Center, Electrical Materials Research Division, Korea Electrotechnology Research Institute, Changwon, South Korea; 2 Department of Electro-functionality Materials Engineering, University of Science and Technology, Changwon, South Korea

## Abstract

The demand for easy-to-use portable electric devices that are combined with essential items in everyday life, such as apparel, has increased. Hence, significant research has been conducted into the development of wearable technology by fabrication of electronic devices with a textile structure based on fiber or fabric. However, the challenge to develop a fabrication method for wearable devices based on weaving or sewing technology still remains. In this study, we have proposed and fabricated a 3-D textile with two electrodes and one spacer in a single sheet of fabric, utilizing a commercial weaving machine. The two electrodes fulfil the role of electron transfer and the spacer between the electrodes circulates electrons and prevents electrical shorting. Hence, the 3-D textile could be applied to a wide range of electrochemical devices. In addition, it is possible to control the textile structure, size and quantity and change the electrode or spacer materials by replacing the thread. We applied the 3-D textile to dye-sensitized solar cells (DSSCs) which has distinctive advantages such as low manufacturing cost, esthetic appearance for interior or exterior application and high power output under relatively weak light illuminations. The 3-D textile DSSCs were fabricated through a continuous process, from manufacturing to encapsulation, using a non-volatile electrolyte and demonstrated a specific power of 1.7% (1 sun, 1.5 A.M.). The 3-D textile DSSCs were electrically connected in parallel and series by twisting, stainless steel wires, which were used as the weft, and a light-emitting diode lamp was turned on using 3-D textile DSSCs connected in series. This study represents the first stage in the development and application of wearable textile devices.

## Introduction

There is increasing demand for easy-to-use portable electronic devices that can be combined with essential items in everyday life. Apparel is an indispensable element of daily life, and the concept of wearable electronics has received significant attention. A number of studies have investigated the development of wearable technology through fabrication of electronic devices, such as energy-harvesting devices^1–11^, energy-storing devices^12–15^, sensors^16–18^ and light-emitting diodes (LEDs)^19–22^, and their integration into textile structures. In particular, wearable electrochemical devices are important for energy harvesting and storage^23–26^.

One approach to fabricating a device in textile form is to integrate all device components into a fiber or wire^6–8,23^; fiber-formed devices are then implemented by inserting or integrating short fiber strands into clothing. However, there are limitations to the length and flexibility of the fibers, as they are encapsulated using polymer tubes. The fibers can also be difficult to weave together densely for forming real textiles, because coated electrode layers can be easily damaged under friction during the weaving process. An alternative approach is the fabrication of textile-based devices by coating the electrode on commercial fabrics or stacking fabrics^9–12,27^, however, weaving or sewing of such fabrics has still not been fully investigated, and further research is needed into weaving or sewing technologies for wearable devices.

We have previously studied textile-formed electrochemical devices for fabrication of a wearable device from real fabrics using sewing and weaving technologies^28,29^. From the perspective of the textile, this process is fully developed, however, complications arise because electrodes were first formed by coating and sintering before combining into one device, and for a simple and continuous process, the textile for the cell should be fabricated first, then the electrodes deposited^30^.

In this study, we propose a 3-D textile with two electrodes and one spacer in a single sheet of fabric using a commercialized weaving machine^31–33^. The 3-D textile could be used for a range of electrochemical devices because it has two electrodes that fulfil the role of electron transfer, and a spacer preventing electrical shorting between the electrodes and circulating the electrons through an electrolyte. It is possible to change the electrode and spacer materials easily and freely by replacing the warp and weft threads in the 3-D textile. The problems of fiber-formed devices can be surmounted because the deposition process occurs after manufacture of the 3-D textile cell. It is also possible to diversify the woven structure, and the size and quantity of the 3-D textile cell, because the weaving process is conducted using a jacquard machine. In addition, multiple 3-D textile cells can be woven simultaneously and then easily cut and separated using scissors.

We applied the 3-D textile cell fabrication method to dye-sensitized solar cells (DSSCs)^34–36^ and confirmed their performance. In a 3-D textile DSSC, one side of the electrode acts as the photoanode, and the other as the counter electrode. The spacer is composed of woven glass fiber filled with electrolyte. Weaving and fabrication the 3-D textile which function as electrochemical device like DSSCs itself is highlighted and differentiated technology comparing to previous reported research like insert a wire which acts as a substrate or device or integration separated textile components for a fabrication device^6–12,28–30^. An easy-to-apply encapsulation technology was used via a lamination machine by infiltration a non-volatile electrolyte^37–39^; this improved the stability of the 3-D textile DSSCs. We demonstrated that the 3-D textile DSSCs could be connected easily in parallel and series by twisting the SUS wires that were used as weft-like conventional DSSCs modules for increasing power output, and confirmed this by turning on an LED lamp connected in series.

## Results and Discussion

**Figure 1:**
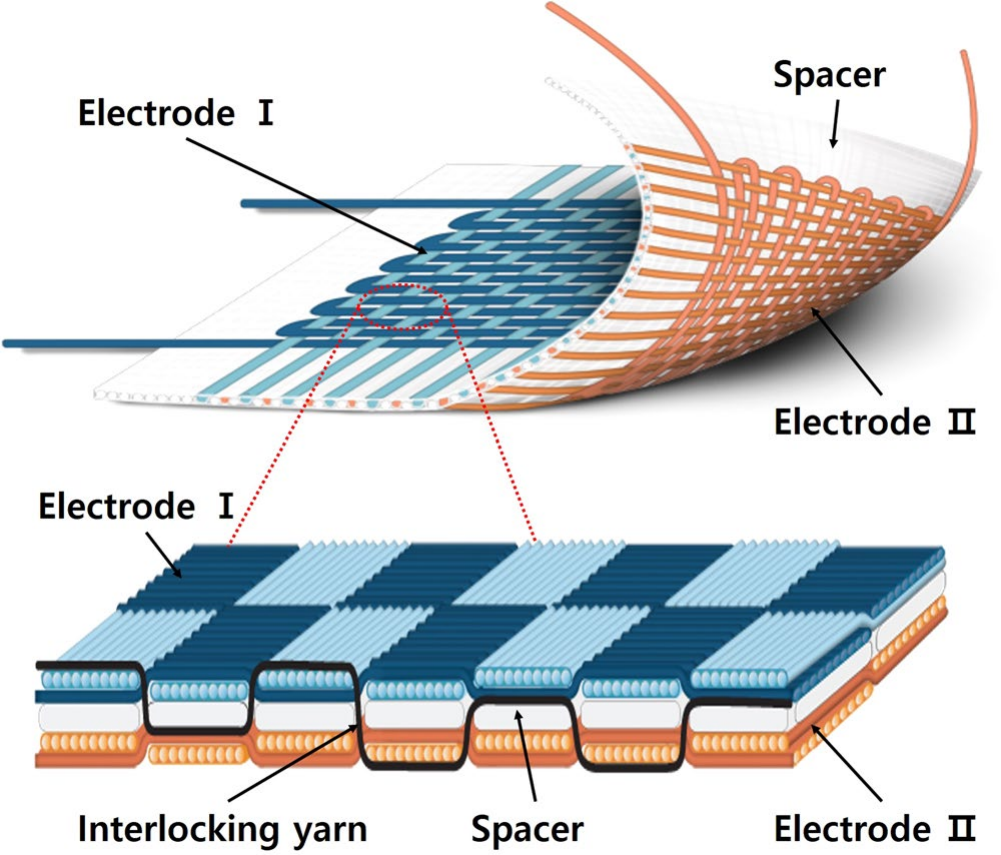
Schematic illustration of a single sheet 3-D textile and cross-sectional magnified view of the 3-D textile.

A 3-D textile is a single sheet of fabric with a configuration involving two electrodes and one spacer, as shown in Fig. 1. The middle part of the 3-D textile has a three-layer structure, both sides are consisted of a woven structure with stainless steel (SUS) wires which is conductive thread so they could act as positive and negative electrodes, and the spacer prevents electrical shorting between the two SUS-woven electrodes. This configuration is identical to that of electrochemical devices, and thus the 3-D textile can be used in a wide range of electrochemical applications.

Specifically, in the 3-D textile, the SUS-woven electrodes had a basket structure (Fig. S1(a)), a flat morphology comparable to plain woven structures (Fig. S1(b)), and greater strength due to the multiple strands of warps and wefts^40,41^. The basket dutch woven structure was used to enhance the strength and density of the fabric and increase the contact surface between the woven electrode and the coated electrode, thereby increasing the number of electrons and the transfer rate (Fig. S1(c)). The spacer should also have a dense structure to prevent electrical shorting of the two woven electrodes. Also considering the redox circuit of the electrolyte, the plain woven structure which have many intersecting point in other terms opening area between warp and weft is suitable to the spacer.

A commercial Jacquard loom was used to fabricate the 3-D textile, which is able to weave complicated patterns. The Jacquard loom consisted of a large number of pistons serving as heddles and strings connecting pistons and warps (Fig. S2(a,b)); the weaving process is conducted as heddles go up and then inserting the weft fiber depending on the pattern of the textile (Fig. S2(c)).

**Figure 2:**
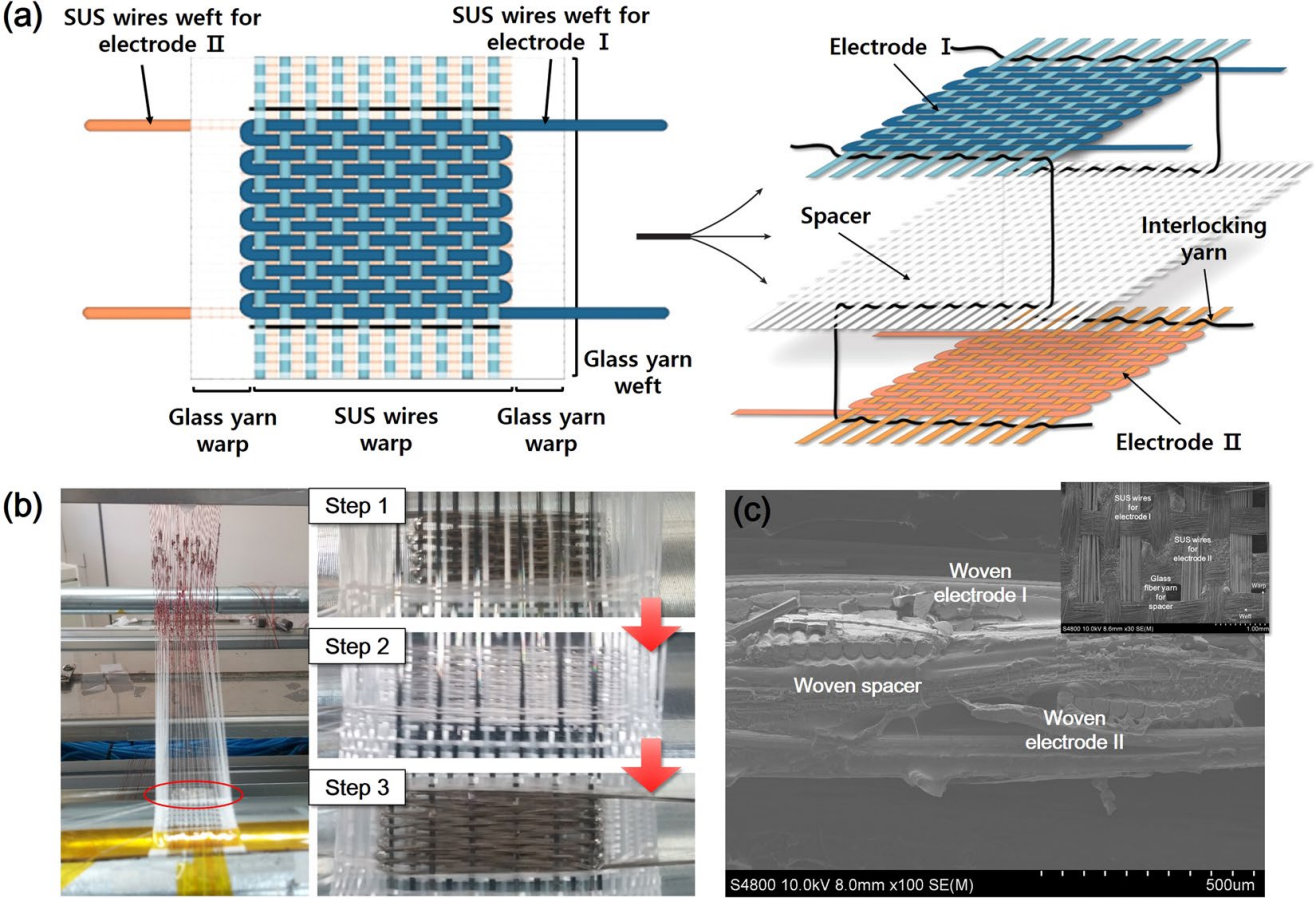
(**a**) Schematic illustration of 3-D textile in planar view and detail of the 3-D structure of the 3-D textile (right illustration). (**b**) Photographs of the weaving process of the 3-D textile with a Jacquard machine. (**c**) Cross sectional scanning electron microscopy (SEM) images of 3-D textile and warp arrangement (inserted image).

Within the 3-D textile, thick glass fiber yarns (22.5 tex) were used as the warp at the edges. In the middle sections of the textile, SUS wires (40 *μ*m) were used for electrodes I and II and thin glass fiber yarns (5.6 tex) were used for the spacer, arranged as warps. The edge sections should be free from warp distortion to facilitate weaving, and should retain their shape even when the textile is cut; hence, the warps in this section were woven into a plain structure using thick glass fiber yarn as a weft. In the main 3-D structure, electrodes I and II and the spacer were woven into three separate layers, and simultaneously tied together by weaving with thin glass fiber yarn to prevent gaps between layers (Fig. 2(a)). Depending on where and how many we place the tied glass fiber yarns, we can control the density of 3-D textile. The warps were connected to the strings of the Jacquard loom and arranged as shown in the left image of Fig. 2(b); the edge of 3-D textile was then woven (red circled image). In step 1, the SUS wires, acting as the warp and weft for electrode I, were woven with a basket dutch structure in the lower layer of the 3-D structure. For the spacer, thin glass fiber yarns as the warp and weft were woven into a plain structure for electrode II (step 2), and then SUS wires acting as the warp and weft for electrode I were woven to create the upper layer of the 3-D structure (step 3). The middle part of the 3-D textile is made up of the 3-D structure shown in Fig. 2(c) in cross section, and at the same time the three layers were tied together without electrical shorting. A straight and ordered arrangement of the warps was confirmed and is shown in the inset image in Fig. 2(c).

The 3-D textile was fabricated from a warp arrangement and direct weaving, so the thread material and weaving pattern of the textile can be changed easily; specifically, the electrode and spacer materials can be changed to create a desired device. In addition, several 3-D textiles can be fabricated simultaneously, and separated by cutting with scissors, as with a conventional fabric (Fig. S3).

**Figure 3:**
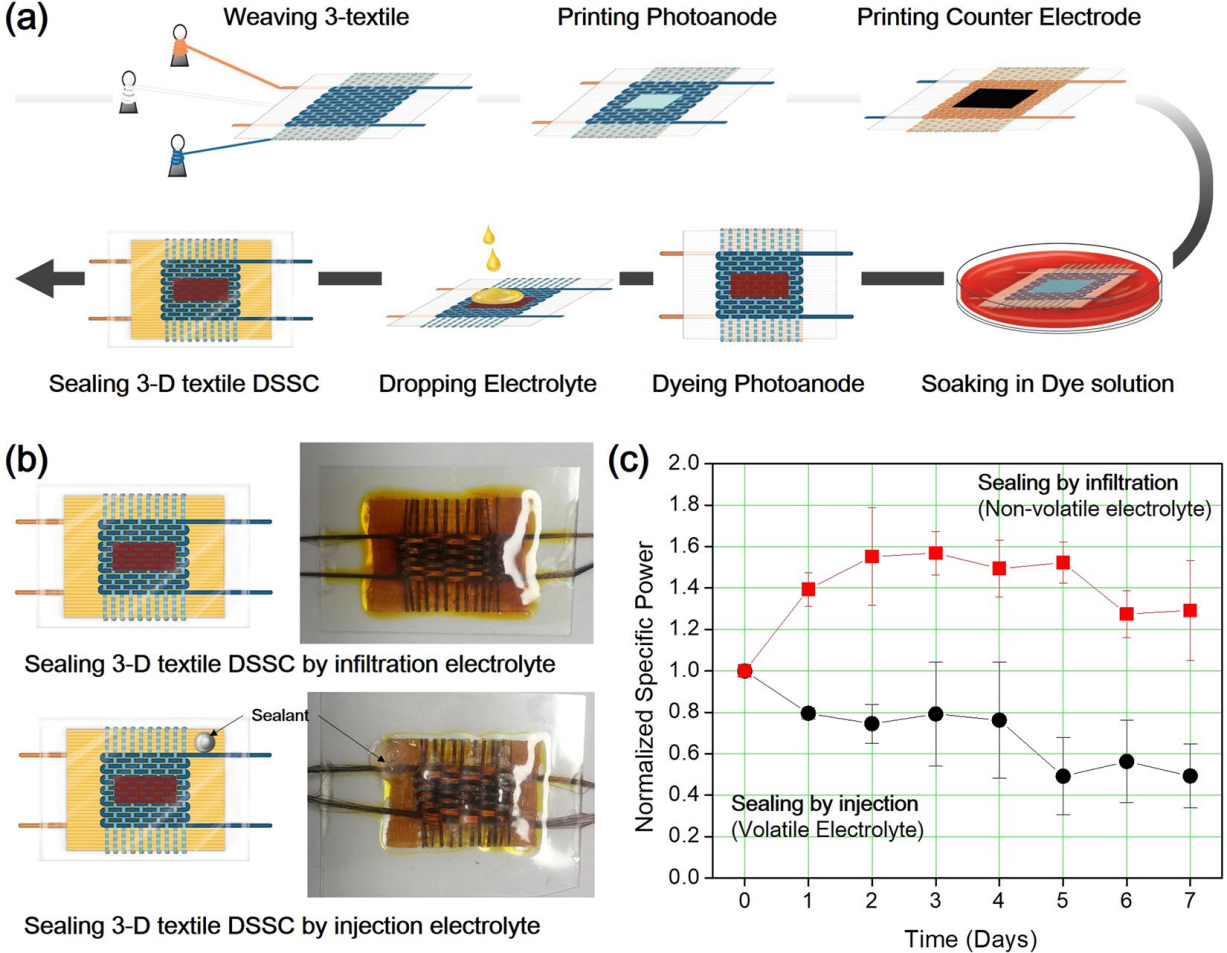
(**a**) Schematic illustration fabrication process of 3-D textile dye-sensitized solar cells (DSSCs). (**b**) Schematic illustration of sealed 3-D textile DSSCs (left column), and photographs (right column) of sealing by infiltration of non-volatile electrolyte (upper images) and injection of volatile electrolyte (below images). (**c**) Normalized specific power of 3-D textile DSSCs sealed by infiltration of non-volatile electrolyte and injection of volatile electrolyte depending on days.

We used our method to fabricate 3-D textile DSSCs. One electrode of the 3-D textile fulfils the role of the photoanode and the other acts at the counter electrode. For the photoanode, TiO_2_ paste was printed on electrode I using the floating printing method^42^, which is suitable for the textile substrate, and then a sintering process was conducted. For the counter electrode, a platinum (Pt) particle coated with activated carbon paste was printed on electrode II. The printing process after fabrication of the 3-D textile prevents cracking or peeling of the deposited electrode, as shown in Fig. S4(a–c) for the photoanode and Fig. S4(d,e) for the counter electrode. After dye-loading on the photoanode, non-volatile succinonitrile (SN)-based electrolyte was infiltrated onto the 3-D textile and then the fabrication process was completed by sealing with polyester (PET) film using the pouching method. Through this process, 3-D textile DSSCs were successfully fabricated and there is a possibility of continuous roll to roll manufacture (Fig. 3(a)).

A non-volatile SN-based electrolyte was used for the sealing process, which can compensate for the significant limitations of liquid electrolytes, which are often unstable and leak easily. Typically, for sealing the DSSCs a hole is made on the substrate for injecting the electrolyte; the hole is then blocked using a sealant. Comparing a typical sealing process with the infiltration electrolyte sealing process, differences were observed in terms of leakage through the sealant and the amount of electrolyte filling required. For the typical sealing process, the electrolyte is filled by injection while extracting the air inside of the cell using a vacuum pump, so it is not possible to be certain that complete filling of the electrolyte has occurred. Furthermore, the remaining air could cause performance loss in the DSSCs. During the electrolyte sealing process, the electrolyte was sufficiently infiltrated on the 3-D textile cell and then the cell was sealed by rolling using the thermal compression bonding method. During rolling, the air was removed and the electrolyte filled simultaneously. The difference in the amount of electrolyte was confirmed visually, as shown in Fig. 3(b). A comparison of DSSC performance, between use of a conventional volatile electrolyte with a typical sealing process, and a non-volatile electrolyte with the infiltration electrolyte sealing process done over time (Fig. 3(c)), indicated that the non-volatile electrolyte had a higher viscosity and hence was stabilized in the cell during the day. The specific power was increased by 30% after 1 day, and then remained stable without any loss over time. In comparison, for the volatile electrolyte with a typical sealing process, the specific power decreased by 20% after 1 day and by 50% after 7 days. This result showed that the infiltration electrolyte sealing process with the non-volatile electrolyte is very stable and not inferior in application.

**Figure 4:**
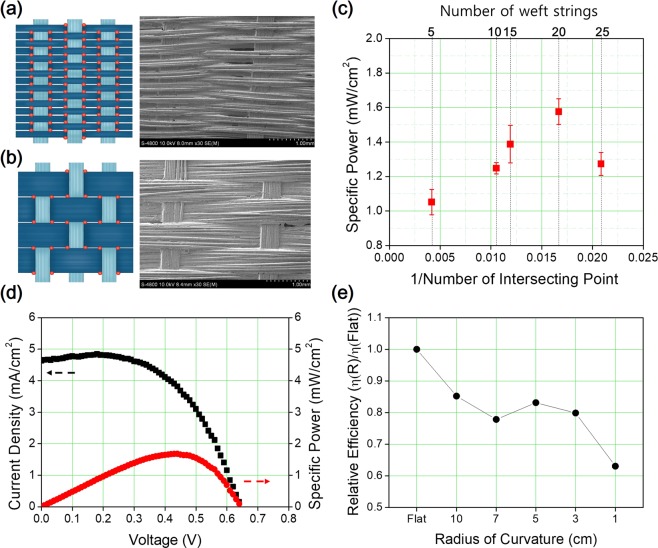
(**a**) Schematic illustration of the surface of a woven electrode with intersecting points (left column) and SEM images (right column) of weaving with 5 strands of wire (**a**) and 20 strands of wire (**b**). (**c**) Average specific power of 3-D textile DSSCs with various numbers of weft strands of the woven electrode. (**d**) Current density–voltage (*J*–*V*) characteristics and power–voltage characteristics of optimized 3-D textile DSSCs. (**e**) Relative energy conversion efficiency to a flat state of 3-D textile DSSCs according to the bending radius of curvature.

The surface of the 3-D textile affects the condition of the printed electrode, which affects the performance of DSSCs by influencing diffusion and recombination. The surface morphology of the woven electrode varies depending on the number of weft strands, which in turn affects the morphology of the printed TiO_2_ layer. The number of wires was changed by increasing the number of strands from 5 to 25, and when weaving the 3-D textile having same width, the number of intersecting points was increased by decreasing the number of strands. The number of intersecting points is significantly different when weaving 5 versus 20 strands, as shown in Fig. 4(a,b), and the specific power increased as the number of intersecting points decreased (Fig. 4(c)). At the point where the warp and weft intersect, a valley is formed; at this intersecting point, the TiO_2_ paste coating was thicker than the surface of the wires of the woven electrode. Recombination occurred in the non-uniform part of the dye-loaded TiO_2_, leading to a reduction in the specific power. In addition, the tension of the weft wires increases as the number of strands increases and the balance of tension between the warp and weft is adjusted during the weaving process. However, when the number of weft wires is above the optimum, the balance between the warp and weft wires is disturbed and weft strands are bundled together. According to this phenomenon, a deeper valley of overlapped wires on the surface is formed, while no uniform TiO_2_ layer is not formed; this decreases the specific power. Detailed images of the woven electrode surface and coated TiO_2_ layer for each configuration are shown in Fig. S5. The optimized 3-D textile DSSCs with non-volatile electrolyte had a specific power of 1.7% under 1-sun illumination, as shown Fig. 4(d) (short-circuit current density, 4.63 mA cm^−2^; open-circuit voltage, 0.64 V; fill factor, 0.56).

This represents good performance for a textile fabricated using a commercial weaving process and a viscous non-volatile electrolyte. The 3-D textile DSSCs exhibited the capacity to bend, and the photovoltaic performance was measured by wrapping around with rods having varying radii of curvature. The performance was maintained at 80% of that of the flat 3-D textile DSSCs, with bending at a 3 cm radius of curvature (Fig. 4(e)). Detailed photographs of this scenario are shown in Fig. S6(a,b). But under bending at 1 cm radius of curvature, the performance of 3-D textile DSSCs decreased to 65% comparing flat of that. At excessive deformation condition, deposited electrode got damaged owing to cracking or separating from the substrate. In addition 1000 times and 2000 times repeating bending test was carried out for the mechanical behavior, the performance was decreased to 60% of initiation performance of 3-D textile DSSCs (Fig. S7). Carbon paste has lack of stability under 200 times bending but it is meaningful to produce counter electrode paste that can be replaced Pt paste.

**Figure 5:**
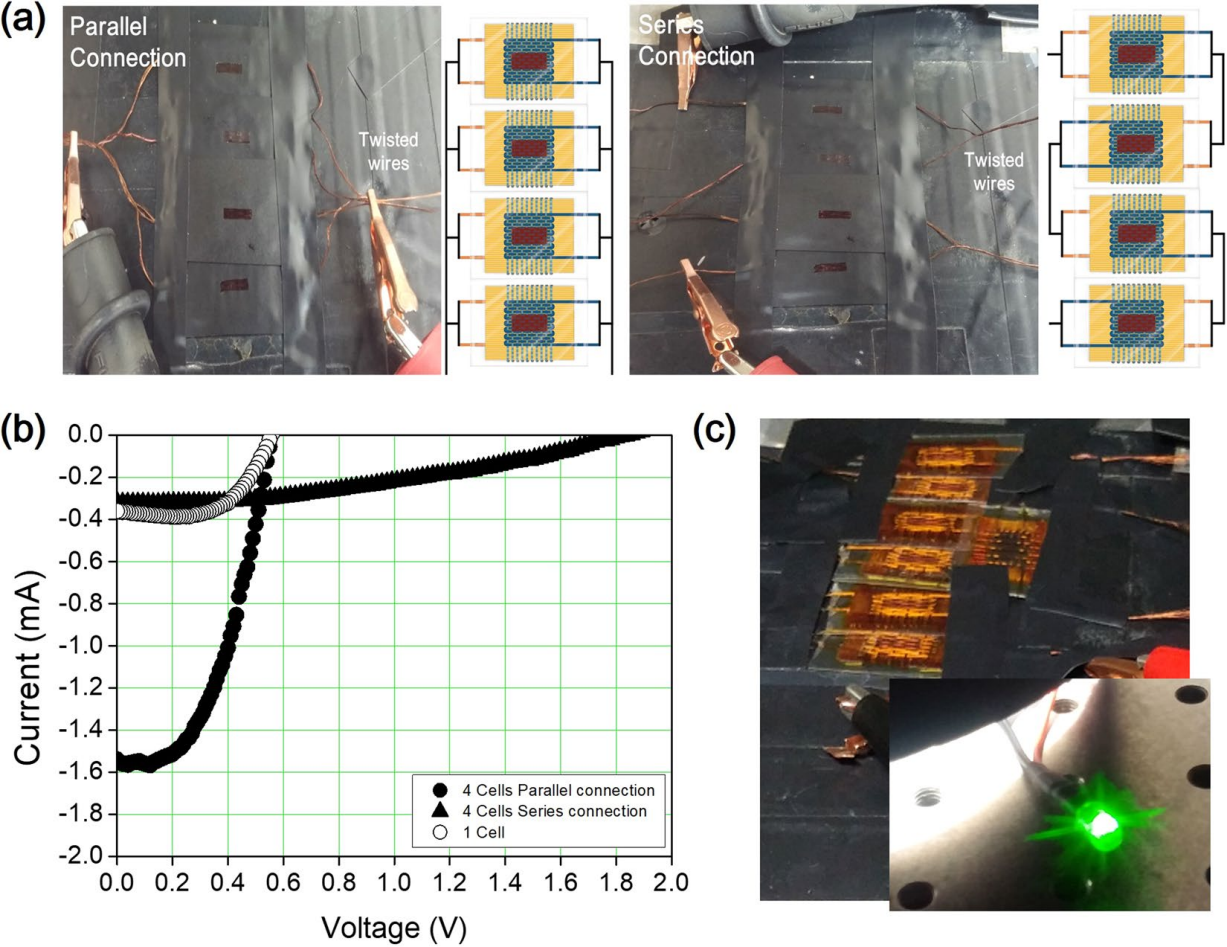
(**a**) Photographs and schematic illustrations of four 3-D textile DSSCs interconnected electrically in parallel and series. (**b**) Current–voltage (*I*–*V*) characteristics of 3-D textile DSSCs and four parallel and series interconnected 3-D textile DSSCs. (**c**) Photographs of seven 3-D textile DSSCs electrically interconnected in series, used to turn on an LED lamp at 1 sun (1.5 A.M.).

To demonstrate the possibility of electrical connections for real-world application, four 3-D textile DSSCs were electrically connected in parallel and series. The SUS wires used as wefts also served as a lead out, and hence the 3-D textile DSSCs could be simply connected by twisting the wires together, as shown in Fig. 5(a), without soldering or the use of grid lines. The performance of the electrically connected 3-D textile DSSCs is shown in Fig. 5(b); for the parallel connection, the short-circuit current increased almost fourfold, and for the series connection, the open-circuit voltage also increased almost fourfold, compared to the single 3-D textile DSSCs. In addition, we confirmed that an LED lamp could be turned on by connecting seven 3-D textile DSSCs in series, as shown in Fig. 5(c). It is significant that 3-D textile devices can be connected in a module simply and easily by twisting or unwinding wires by hand, and this result demonstrates the advantages of the textile. In addition to maximizing the advantages of using a Jacquard weaving machine, it is expected that by increasing the number of warp arrays for one line of 3-D textile, the number of 3-D textile devices can be increased exponentially in one fabrication run. It is therefore possible to connect several 3-D textile devices electrically at the same time with weaving.

In this study, we fabricated a 3-D textile and confirmed its potential by applying the fabrication method to DSSCs. This 3-D textile can be applied to a wide range of electrochemical devices by changing the electrode or spacer materials, or coating the woven electrode with electrode paste. Also, through the twisting connection method, it is likely that this method is close to commercial application for the production of wearable textile devices, because electrical connection of 3-D textile devices can be achieved during mass production simultaneously with the weaving process using the Jacquard machine. We anticipate that our research, which demonstrates the process and fabrication of a textile, will contribute significantly to the development of textile-formed devices that can be integrated into apparel.

## Summary

A 3-D textile, with two electrodes for electron transfer and one spacer for circulating electrons and preventing shorting between the electrodes, was fabricated using a Jacquard weaving machine. This structure can be applied to a wide range of electrochemical devices. In addition, by adapting the weaving technology, the 3-D textile cell could be diversified through changes in the woven structure, size or electrode or spacer materials, and large numbers of devices can be fabricated and easily cut as required.

We applied the 3-D textile to DSSCs that is possible to applicate for wearable electronic device with light-weight, flexible and outstanding outlooks characteristics. 3-D textile DSSCs have been demonstrated a specific power of 1.7% under 1 sun, 1.5 A.M. illumination and electrical connection in parallel and series; an LED lamp was turned on by a series connection of seven 3-D textile DSSCs. Our study represents the first stage in developing wearable and portable textile electronic devices using weaving technology for commercial application.

## Experimetal Details

### Fabrication of the 3-D textile

Middle part of single sheet of 3-D textile, two electrodes and one spacer are consisted. A 304 SUS wire of 40 *μ*m diameter (Wooyang) was used as the warp and weft fabric for electrodes I and II. Two electrode parts has basket dutch woven structure utilizing multiple strands of SUS wires as warps and wefts. Glass fiber yarns of 22.5 tex (D450 1/2; Hyunmin Fiber) and 5.6 tex (D900; Hyunmin Fiber) were used as the warp and weft for each border section and the spacer, respectively. Considering the role of spacer, plain woven structure was applied to spacer part. Before weaving, the SUS wires were rinsed with acetone, ethanol and deionized (DI) water under sonication and dried. A jacquard loom with many pistons (Daesung High-tech) was used for weaving.

### Fabrication of the 3-D textile DSSCs

TiO_2_ paste containing 20 nm TiO_2_ nanoparticles (EnB Korea) was printed on one side of woven electrode for the photoanode using the floating printing method. The woven electrode was masked using 3 M tape, then heat-treated at 480 °C for 1 h in air. The active area of the photoanode was 8 mm × 3 mm. For fabrication of the counter electrode paste, 50 mg of activated carbon (AC.; Kuraray Chemical) and 50 mM H_2_PtCl_6_∙6H_2_O (Sigma Aldrich) were dispersed in 30 ml water and ultrasonicated for 30 min; then, the solution was stirred for 1 h. A volume of 40 ml of NaBH_4_ solution was added to the carbon suspension with stirring for 6 h. The solid product obtained through filtering was dried at 250 °C for 1 h. Pt/AC powder was mixed with carboxylmethyl (CMC) cellulose (Sigma Aldrich) and DI water in a weight ratio of 9:1:40 (Pt/AC:CMC:DI water). A Pt/AC paste was printed using the doctor blade method on the other side of the woven electrode of the 3-D textile. The 3-D textile was then immersed in a 0.3 mM ethanol solution (Sigma Aldrich) of N719 dye (Solaronix) at room temperature for 20 h for dyeing.

After printing and dyeing, non-volatile electrolyte (succinonitrile (SN)-based electrolyte) was infiltrated on the 3-D textile cell and encapsulated with PET-EVA film using a hot roller machine (SINDOH Commerce). For comparison with the injection encapsulation process done using a volatile electrolyte (acetonitrile (AN)-based electrolyte), a hole was made by micro-drilling on the film and electrolyte was injected through the hole. Then, the hole was sealed using a vacuum sealant. The SN-based electrolyte was composed of 1.38 M 1-ethyl-3-methylimidazolium iodide (EMII), 0.07 M iodine, 0.13 M guanidine thiocyanate, 0.85 M 4-tert-butylpyridine, and 0.7 M lithium iodide in SN. The AN-based electrolyte was composed of 0.6 M 1-butyl-3-methylimidazolium iodide (BMImI), 0.03 M iodine, 0.1 M guanidine thiocyanate, 0.5 M 4-tert-butylpyridine, and 0.1 M lithium iodide dissolved in 3-methoxypropionitrile and AN at a 2:8 volume ratio.

### Characteristics

Field-emission scanning electron microscopy (FE-SEM; Hitachi S4800) was performed to observe the surface and cross section of the 3-D textile DSSCs. The energy conversion performances of the 3-D textile DSSCs were evaluated using a solar simulator (model Sun 2000; Abet Technologies, 1000 W Xe source; Keithley 2400 source meter) under 1.5 AM, 1 sun conditions, calibrated by a KG-3 filter and National Renewable Energy Laboratory (NREL)-certified reference cell with a mask (7 × 2 mm). Electrical connection in parallel and series was conducted for four 3-D textile DSSCs and an LED lamp was turned on by seven 3-D textile DSSCs.

## Supplementary information

Supplementary Information
