# Supplementary material for: Three-Dimensional Textile Platform for Electrochemical Devices and its Application to Dye-Sensitized Solar Cells
Source: Sci Rep. 2019 Feb 20;9:2322. doi: 10.1038/s41598-018-38426-1 (PMC6382877; doi:10.1038/s41598-018-38426-1)
Supplement: Supplementary file 1 — Supplementary Information [file 41598_2018_38426_MOESM1_ESM.docx]

**Supplementary Information**

**Three-Dimensional Textile Platform for Electrochemical Devices and its Application to Dye-Sensitized Solar Cells**

**Min Ju Yun^1^, Yeon Hyang Sim^1,2^, Seung I. Cha*^1,2^, Seon Hee Seo^1^, Dong Y. Lee^1,2^**

**1. Nano Hybrid Technology Research Center, Creative and Fundamental Research Division,**

**Korea Electrotechnology Research Institute**

**2. Department of Electro-functionality Materials Engineering, University of Science and Technology**

**
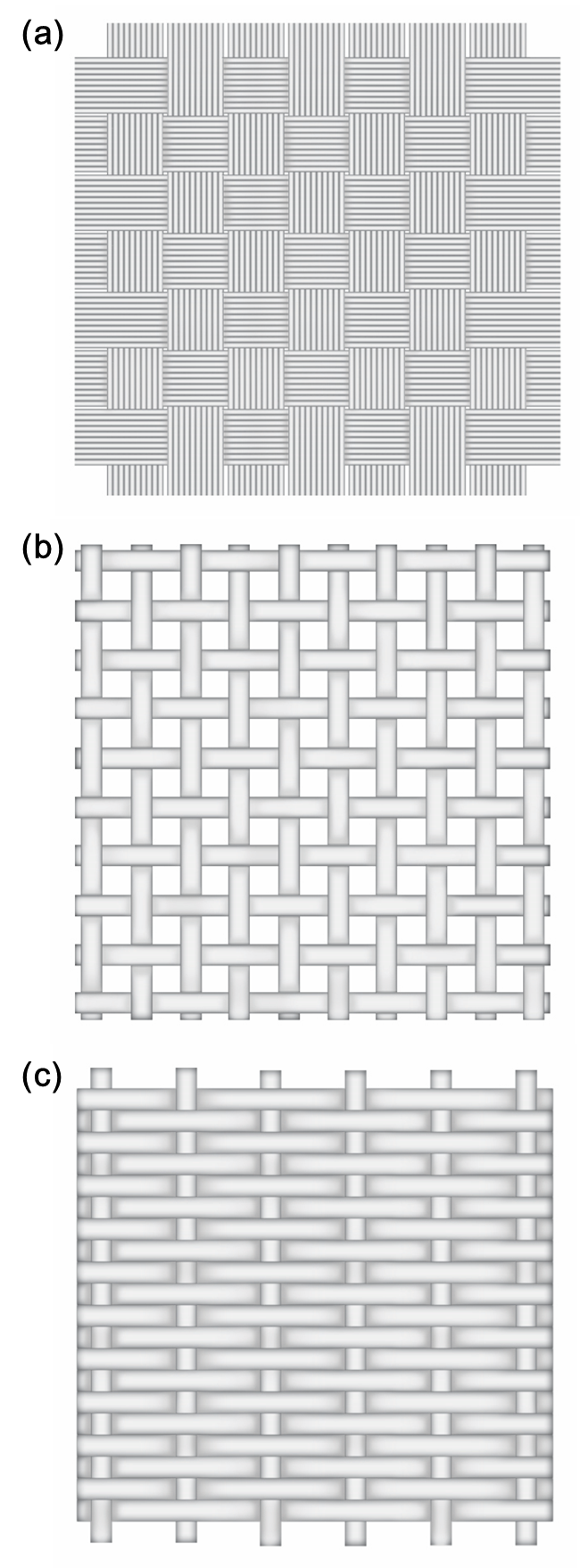
**

**Figure S1.** Schematic illustration of (a) basket dutch, (b) plain and (c) plain dutch woven structure.

**
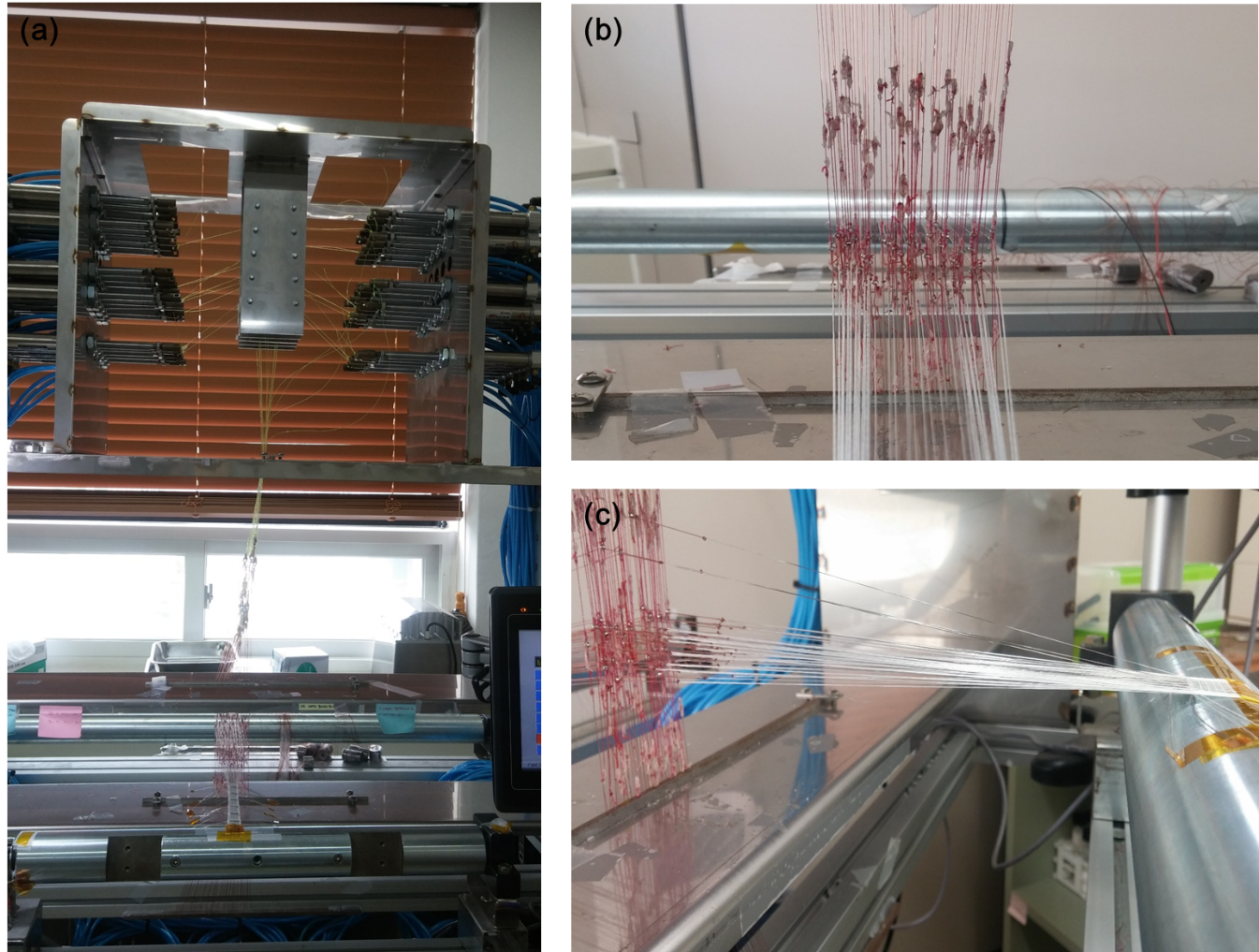
**

**Figure S2.** Photographs of (a) jacquard weaving machine, (b) strings which is connected to pistons and warp threads and (b) going up the heddles and warp threads.


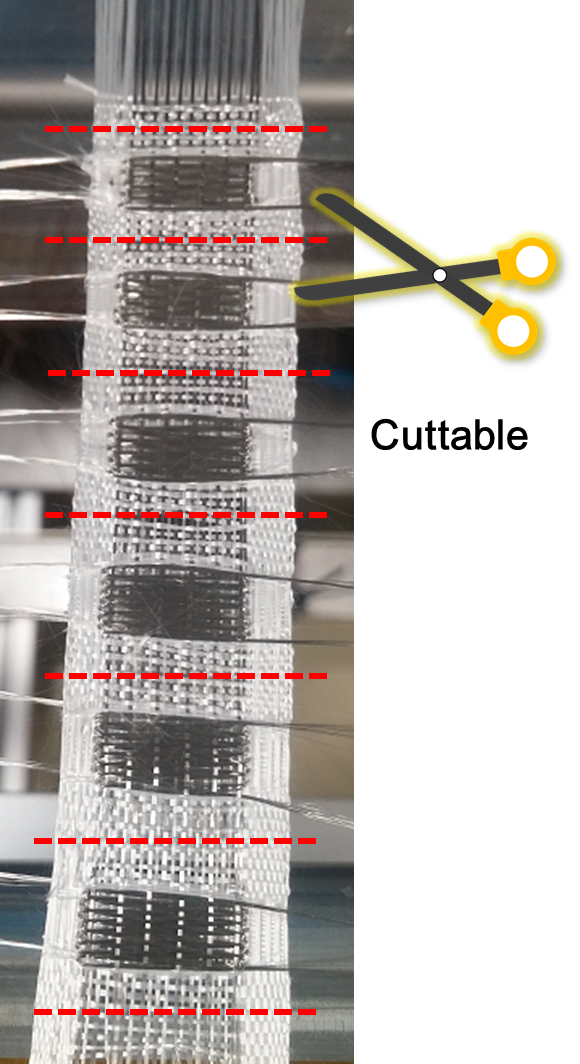


**Figure S3.** Photograph of multiple 3-D textile cells woven into one line.


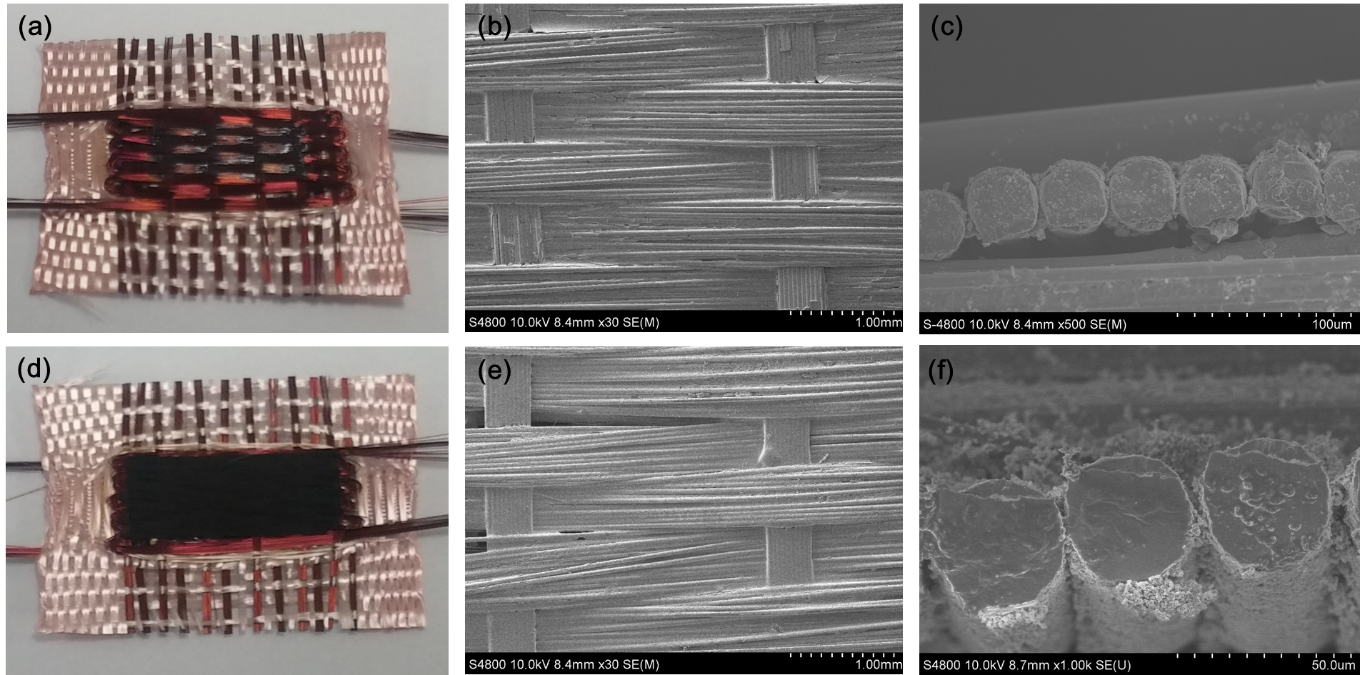


**Figure S4**. (a) Photograph of 3-D textile DSSC deposited photoanode and scanning electron microscopy (SEM) images of (b) plain and (c) cross section view. (d) Photograph of 3-D textile DSSC deposited counter electrode and scanning electron microscopy (SEM) images of (e) plain and (f) cross section view.


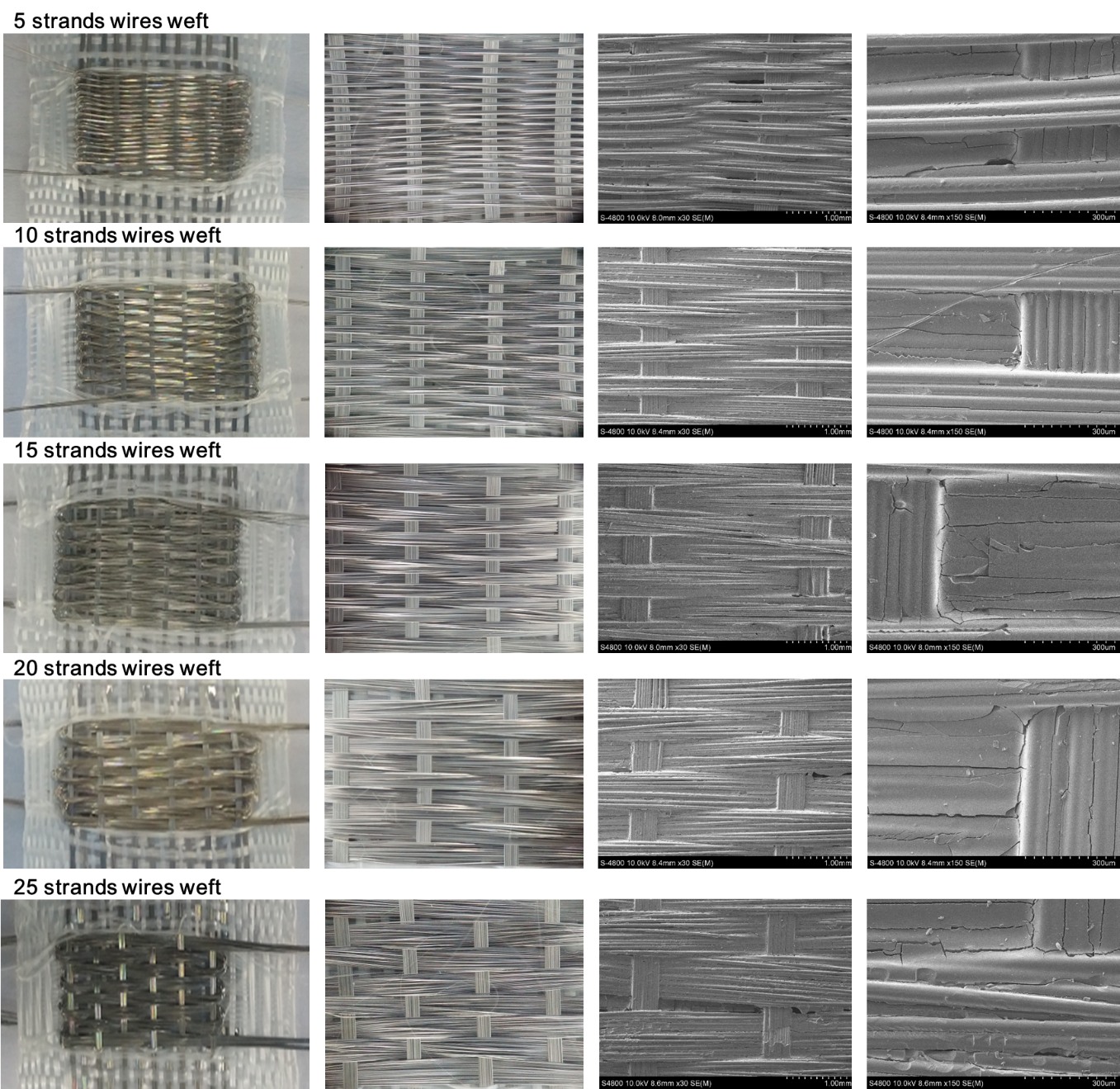


**Figure S5.** Photographs of woven electrode of 3-D textile cell and SEM images of deposited photoanode on woven electrode depending on number of intersecting points between warps and wefts.


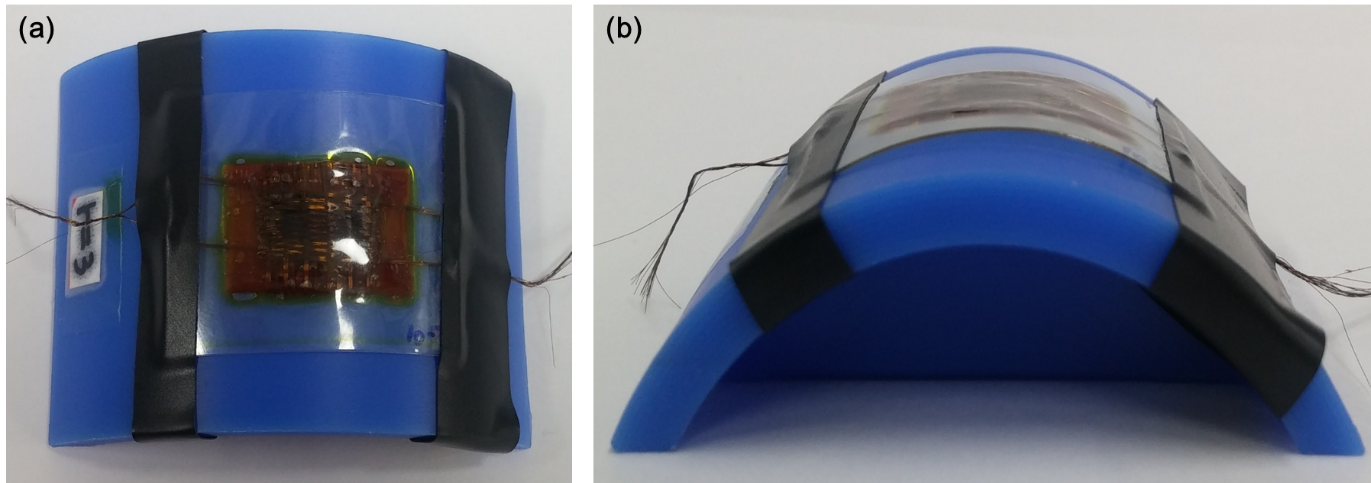


**Figure S6.** Photographs of curved 3-D textile DSSC wrapped on 3cm diameter of rod in (a) plain and (b) cross section view.

Figure S7. The relative energy conversion efficiency of flat state of 3-D textile DSSCs according to repeating bending deformation.
